# Genome-Wide Association and Mendelian Randomization Analysis Reveal the Causal Relationship Between White Blood Cell Subtypes and Asthma in Africans

**DOI:** 10.3389/fgene.2021.749415

**Published:** 2021-12-02

**Authors:** Opeyemi Soremekun, Chisom Soremekun, Tafadzwa Machipisa, Mahmoud Soliman, Oyekanmi Nashiru, Tinashe Chikowore, Segun Fatumo

**Affiliations:** ^1^ The African Computational Genomics (TACG) Research Group, MRC/UVRI and LSHTM, Entebbe, Uganda; ^2^ H3Africa Bioinformatics Network (H3ABioNet) Node, Centre for Genomics Research and Innovation, NABDA/FMST, Abuja, Nigeria; ^3^ Department of Medicine, University of Cape Town, Groote Schuur Hospital, Cape Town, South Africa; ^4^ The Department of Pathology and Molecular Medicine, Population Health Research Institute (PHRI), Michael G. DeGroote School of Medicine, McMaster University, Hamilton, ON, Canada; ^5^ Molecular Bio-Computation and Drug Design Laboratory, School of Health Sciences, University of KwaZulu-Natal, Westville Campus, Durban, South Africa; ^6^ Faculty of Health Sciences, Sydney Brenner Institute for Molecular Bioscience, University of the Witwatersrand, Johannesburg, South Africa; ^7^ MRC/Wits Developmental Pathways for Health Research Unit, Department of Paediatrics, Faculty of Health Sciences, University of the Witwatersrand, Johannesburg, South Africa; ^8^ Department of Non-Communicable Disease Epidemiology, London School of Hygiene and Tropical Medicine, London, United Kingdom

**Keywords:** white blood cell traits, asthma, metanalysis, Mendelian randomization, fine mapping

## Abstract

**Background:** White blood cell (WBC) traits and their subtypes such as basophil count (Bas), eosinophil count (Eos), lymphocyte count (Lym), monocyte count (Mon), and neutrophil counts (Neu) are known to be associated with diseases such as stroke, peripheral arterial disease, and coronary heart disease.

**Methods:** We meta-analyze summary statistics from genome-wide association studies in 17,802 participants from the African Partnership for Chronic Disease Research (APCDR) and African ancestry individuals from the Blood Cell Consortium (BCX2) using GWAMA. We further carried out a Bayesian fine mapping to identify causal variants driving the association with WBC subtypes. To access the causal relationship between WBC subtypes and asthma, we conducted a two-sample Mendelian randomization (MR) analysis using summary statistics of the Consortium on Asthma among African Ancestry Populations (CAAPA: *n*_cases_ = 7,009, *n*_control_ = 7,645) as our outcome phenotype.

**Results:** Our metanalysis identified 269 loci at a genome-wide significant value of (*p* = 5 × 10^−9^) in a composite of the WBC subtypes while the Bayesian fine-mapping analysis identified genetic variants that are more causal than the sentinel single-nucleotide polymorphism (SNP). We found for the first time five novel genes (*LOC126987*/*MTCO3P14*, *LINC01525*, *GAPDHP32*/*HSD3BP3*, *FLG-*AS1/HMGN3P1, and *TRK-CTT13-*1/MGST3) not previously reported to be associated with any WBC subtype. Our MR analysis showed that Mon (IVW estimate = 0.38, CI: 0.221, 0.539, *p* < 0.001), Neu (IVW estimate = 0.189, CI: 0.133, 0.245, *p* < 0.001), and WBCc (IVW estimate = 0.185, CI: 0.108, 0.262, *p* < 0.001) are associated with increased risk of asthma. However, there was no evidence of causal relationship between Lym and asthma risk.

**Conclusion:** This study provides insight into the relationship between some WBC subtypes and asthma and potential route in the treatment of asthma and may further inform a new therapeutic approach.

## Introduction

Hematological cells play critical roles in protecting the host organism against immune assault (Li et al., 2013). Dysregulation or aberration within the hematopoietic system has been implicated in several diseases, and this could as well serve as prognostic markers. For example, an aberration in leukocytes could be an indicator of lymphoma, leukemia, heart failure, polycythemia, and hypertension (Buttari et al., 2015). White blood cells (WBCs) play a major role in both innate and adaptive immune systems, serving as the primary defense system against foreign assaults. Due to the role they play in defense and immunity, they are used as biomarkers for detecting inflammation (Gopal et al., 2012). High WBC count has been linked to the pathogenesis of different disease conditions such as cardiovascular disease and cancer (Keller et al., 2014). WBCs are categorized into five subtypes based on their functions and morphology: basophils (Baso), eosinophils (Eos), lymphocytes (Lym), monocytes (Mon), and neutrophils (Neu). WBC is an averagely heritable trait, with *h* ^2^ estimates between 0.14 and 0.40 across all the WBC types (Chinchilla-Vargas et al., 2020).

Asthma is a major health problem in the world, and scientific advances in the last two decades have improved our understanding and means of managing it effectively (Bateman et al., 2008). Studies have estimated the global prevalence of asthma to be 1%–18% (Masoli et al., 2004; Bateman et al., 2008; To et al., 2012). Despite its burden in Africa, asthma is classified as one of the neglected diseases with an estimated average of 12% (Asher et al., 2006; Adeloye et al., 2013; Hodonsky et al., 2020).

Several genome-wide association studies that have been carried out on WBC have reported more than 600 associated loci (Yang et al., 2007; Ferreira et al., 2009; Lo et al., 2011; Okada et al., 2011; Reiner et al., 2011; Keller et al., 2014; Astle et al., 2016). Despite the genetic diversity inherent within the African population, most WBC GWAS have been carried out in European- or East Asian-ancestry populations.

Mendelian randomization (MR) is a method that uses genetic proxies as instrumental variables and has been employed to investigate causal inference between an exposure and outcome phenotype (Fall et al., 2015).

Therefore, in this study, we performed an ancestry-specific metanalysis of GWAS of WBC and the five subtypes, Baso, Eos, Lym, Mon, and Neu in participants from two cohorts. The main aim of this study is to identify novel loci and signals associated with WBC traits and assess the causal relationships between these traits and asthma using SNPs as genetic instruments. Findings from this study may provide some biological and pathogenic insight into hematological disorders within the African population.

## Methods

### Study Population

#### APCDR Cohort

The African Partnership for Chronic Disease Research (APCDR) is an organization set out to advance collaboration of epidemiological and genomic research of non-communicable diseases in sub-Saharan Africa. The APCDR cohort comprises four studies: the Ugandan Genome Resource (UGR), the Durban Diabetes Study (DDS), the Durban Case–Control Study (DCC), and the Africa America Diabetes Mellitus Study (AADM).

DDS is a population-based study carried out among non-pregnant black African individuals resident in eThekwini municipality in Durban South Africa from November 2013 to December 2014 (Hird et al., 2016). The survey, which had 1,165 individuals, combined different socioeconomic metrics with anthropometric measurements for infectious and non-communicable diseases. A detailed description of this study population and study design can be accessed in the paper (Hird et al., 2016).

The General Population Cohort (GPC) is a two-phase sample collection study composed of UGWAS (the first sample collected) and UG2G (second samples collected). GPC is a population-based study comprising approximately 22,000 residents of Kyamulibwa located in the southwestern part of Uganda. The goal of the study was to unravel the epidemiology and genetic drivers of non-communicable and communicable diseases using an Afrocentric population (Gurdasani et al., 2019).

The Diabetes Case–Control study is a study consisting of individuals of Zulu ancestry residing in KwaZulu-Natal, aged 40 and above that have diabetes, recruited in a tertiary health facility in Durban. A total of 1,600 individuals were recruited for this study.

The AADM study is a genetic epidemiological study of individuals with type 2 diabetes and associated diseases in Africans; this study has extensively been described in other studies (Rotimi et al., 2004; Adeyemo et al., 2015).

#### The Blood Cell Consortium Cohort

BCX2 is a consortium comprising of trans-ethnic data of blood cell traits of 746,667 individuals from five different ancestries. From the BCX2, we retrieved WBC traits from individuals with African ancestry composed of data pulled from the BioMe™ BioBank Program, Cardiovascular Health Study, Genetic Epidemiology Research on Adult Health and Aging, Jackson Heart Study, The Multi-Ethnic Study of Atherosclerosis, and the UK Biobank African ancestry. The same units were used across the WBC traits in all the cohorts after inverse transformation [WBCc (10^9^/L), Mon (10^9^/L), Neu (10^9^/L), Eos (10^9^/L), Baso (10^9^/L), and Lym (10^9^/L)]. To obtain the pool effects of all the studies involved in the BCX2, an inverse variance-weighted fixed-effect meta-analyses was performed with the aid of GWAMA. For the study level association analysis, an additive genetic model of association was used to determine SNP association while a linear mixed-effect model was employed to factor in cryptic relatedness.

### Hematological Phenotype

White blood and red blood indices of all participants in the DDS cohort were determined using a SYSMEX XT-2000i machine. For the UGR cohort, an 8.5-ml vacutainer was used to collect the venous blood while a 6-ml EDTA bottle was used to collect whole blood. The collected blood samples were stored at a temperature of 4–8°C. For hematological analysis, a swing bucket centrifuge was used to centrifuge the samples at 1,000–13,000 RCF (*g*) for 10 min.

### Genotyping, Quality Control, and Imputation

Genotyping and quality control techniques have been described for each population previously (Gurdasani et al., 2019). Briefly, the DDS samples were genotyped on Illumina HumanOmni Multi-Ethnic GWAS/Exome Array employing the Infinium Assay. Illumina GenCall algorithm was used for genotype calling. The 5,000 GPC samples were genotyped on the Illumina HumanOmni 2.5M BeadChip array (Table 1). Quality control of the DDS cohort took into consideration the following criteria: exclusion of SNPs with heterozygosity > 4 SD from the mean, called proportion <97%, and sex check fails (*F* statistic >0.2 for women and <0.8 for men). Likewise, SNP QC ensured that called proportion was <−97%, relatedness (IBD >0.90), and Hardy–Weinberg disequilibrium (*p* < 10^−6^). Imputation was done on pre-phased data with IMPUTE2 using a merged reference label of the whole genome sequence data from the African Genome Variation Project. Affymetrix Axiom PANAFR SNP array was used to genotype the AADM data as described previously (Adeyemo et al., 2015). Genotyping, imputation, and quality control of the African ancestry cohort of the BCX2 has been described somewhere else (Chen et al., 2020a).

**TABLE 1:** Total number of samples analyzed in APCDR and BCX2.

Traits	APCDR	BCX2	Total
WBC Count	2,741	15,061	17,802
Lymph count	2,681	13,477	16,158
Mono count	2,681	13,471	16,152
Eos count	2,671	11,615	14,286
Baso count	2,681	11,502	14,183
Neu count	2,671	13,476	16,147

^*^African Partnership for Chronic Disease Research (APCDR); Blood Cell Consortium Cohort (BCX2); Mon, Monocytes; Neu, Neutrophil count; WBC, White blood cell count (WBCC).

### Meta-Analysis

Prior to metanalysis, all summary statistics data were manually checked for integrity and accuracy (i.e., summary statistics downloaded have the required variables and are appropriately labeled). Some quality control measures were applied to the summary statistics, SNPs in each cohort having MAF >0.05 were selected. The SNP association *p* values from the summary statistics were meta-analyzed with the aid of GWAMA (Genome-Wide Association Meta-Analysis) (Morris, 2010). We further applied genomic control, and Manhattan plots and quantile–quantile plots were plotted for the meta-analyzed result.

### Statistical Fine Mapping

Following our result output from meta-analysis, we used fine-mapping analysis to pick out possible causal SNPs for the locus ±250 kb of all the lead SNPs, using a Bayesian approach (Maller et al., 2012). The *Z* score was used to calculate the Bayes factor for each SNP denoted as $BF_{i}$ , given by

$$BF_{i}=e^{\left[\frac{Z*Z-\log\left(K\right)}{2}\right]}$$

Where *K* is the number of studies. The posterior probability of driving the association for each SNP was computed by

$$Posterior\;probability\;\;=\;\;\;\;\;\frac{BF_{i}}{\sum_{j}BF_{j}}$$

Where the summation in the denominator is over all SNPs at the locus.

Ninety-nine percent credible set sizes were derived by sorting all the SNPs according to their posterior probability $BF_{i}$ at the locus from the highest to the lowest, and then counting the number of SNPs needed to attain a cumulative posterior probability that is greater than or equal to 0.99. Index SNPs accounting for more than 50% posterior probability of driving the WBC association at a given signal were defined as high confidence.

### Identification of Potential Novel Variants and Locus Definition

Chen et al. (2020a) had previously performed a trans-ethnic and ancestry-specific GWAS of blood cell traits using 746,667 individuals (Chen et al., 2020b). To determine if the variants derived from our study are novel, and perhaps identify novel variants, we checked if any of our loci was reported or fall within ±250 kb window of those identified by Chen et al. A locus is further defined as ±250 kb around the significant SNPs (*p* < 5 × 10^−09^).

### Two-Sample Mendelian Randomization

We performed a two-sample Mendelian randomization analysis using the R-based MR package Mendelian Randomization (Yavorska and Burgess, 2017). To identify independent genetic instruments, we used significant SNPs (*p* < 5 × 10^−09^) in the summary statistics of Lym, Mon, Neu, and WBCc derived from the metanalysis of APCDR and BCX2 summary statistics. We ensured that the instruments selected were not in LD with each other so that their impact on the exposure and outcome are uncorrelated. This was achieved by using *r* ^2^ < 0.001 and a 250-kb clumping upstream and downstream of the lead SNPs. We used asthma as our outcome phenotype. These data were selected from the Consortium on Asthma among African Ancestry Populations (CAAPA; 7,009 cases and 7,645 controls) (Daya et al., 2019). Causal estimates were calculated based on IVW and sensitivity analysis was carried out using MR-Egger and median-weighted methods. For each trait, we used *Q*-statistics to account for heterogeneity in the instruments and also excluded SNPs that may show pleiotropy. Proxy SNP for each missing SNP was obtained from LDProxy (Machiela and Chanock, 2015).

### Functional Analysis

Functional analysis of SNPs identified by the metanalysis was carried out using FUMA (Watanabe et al., 2017). Independent SNPs in linkage disequilibrium or within the same genomic location with the sentinel SNPs were separated. A *p*-value cutoff of *p* < 5 × 10^−09^ and 1,000 G Phase3 AFR reference panel were used. We carried out other functional analysis such as eQTL tissue expression, pathway enrichment analysis, and biological process to get more insights into the functionality of the loci identified. eQTL mapping was performed by mapping SNPs to genes up to 1 Mb and using the Blood eQTL. We used GeneCards (Stelzer et al., 2016) to determine the functions of the gene. GWAS catalogue and Open Targets were used to identify any previously associated phenotypes of the lead SNPs. Annotation of all the genes identified in this study was done using NCBI’s Genome data viewer. Pathway analysis of the identified loci was performed using Enrichr; Enrichr is an integrative web-based server that facilitates the visualization of the functional characteristics of a gene set. Enrichr is available online at http://amp.pharm.mssm.edu/Enrichr.

## Results

### Description of Study

The total samples analyzed for each WBC trait are shown in Table 1. WBCc is the trait with the highest number of individuals and Baso has the lowest samples analyzed.

### Metanalysis of WBC From APCDR and BCX2

**FIGURE 1:**
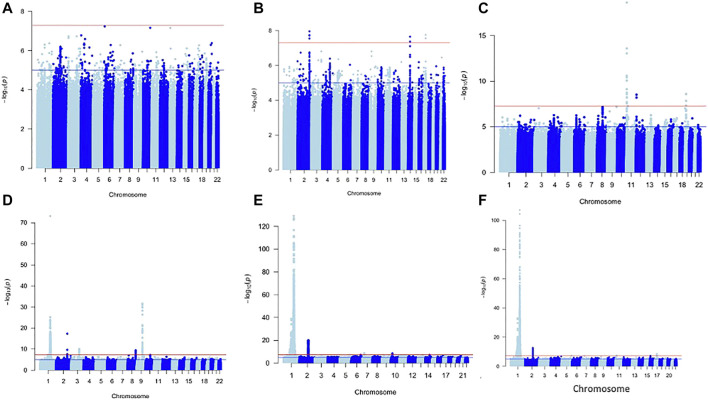
Manhattan plot of metanalysis results of Baso **(A)**, Eos **(B)**, Lym **(C)**, Mon **(D)**, Neu **(E)**, and WBC **(F)**.

For each of the WBC trait subtypes: Lym: 13 SNPs, Mon: 680 SNPs, Neu: 5,308 SNPs, and WBCc: 4,462 SNPs attained genome-wide significance (*p* < 5 × 10^−09^) (Figure 1). No SNPs in the Baso and Eos were significant at *p* < 5 × 10^−09^. Using a genomic distance of ±250 kb, we identified 4, 34, 124, and 108 lead SNPs within different loci in Lym, Mon, Neu, and WBCc, respectively, at *p* < 5 × 10^−09^ [Supplementary Table S1 (ST1–ST4)]. We examined if the sentinel SNPs were within ±250 kb of SNPs reported in the Chen et al. study. When compared to this, all the SNPs in Lym fall with ±250 kb of those previously reported, while only eight SNPs in Mon, 38 SNPs in Neu, and 13 SNPs in WBCc were unique. However, 4 SNPs out of the 8 SNPs unique in Mon, 3 out of 38 in Neu, and 1 out of 13 in WBCc have been previously identified to be associated with WBC traits when queried on the GWAS catalogue (Buniello et al., 2019) and Open Targets (Koscielny et al., 2017). Two SNPs (rs1103700 and rs6693634 mapped near *RPS10P8/CD1A* and *UHMK1/UQCRBP2*) are common in Mon and Neu.

### Fine Mapping

**FIGURE 2:**
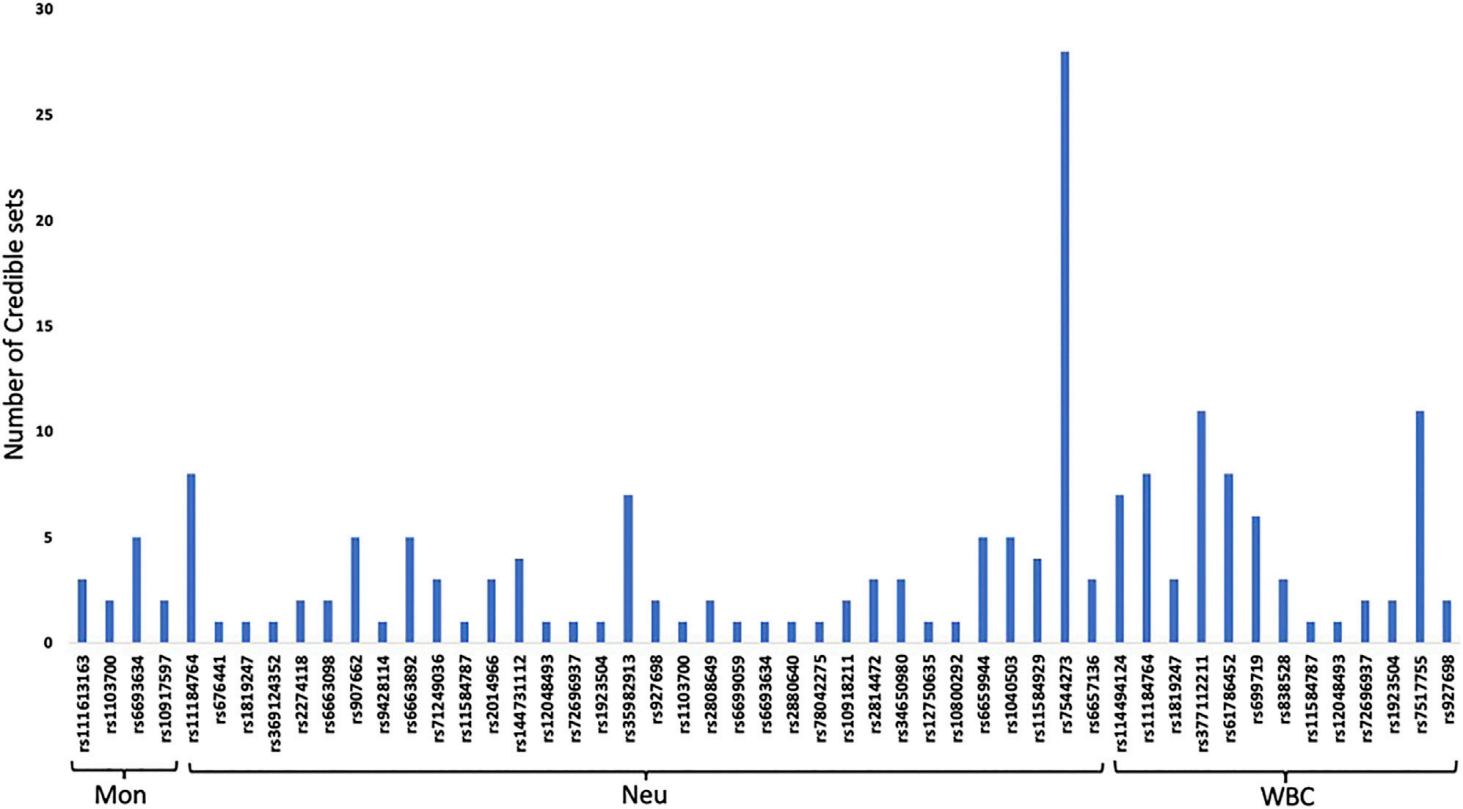
99% credible set of SNPs that jointly make 99% of the posterior probability of driving each WBC traits.

**FIGURE 3:**
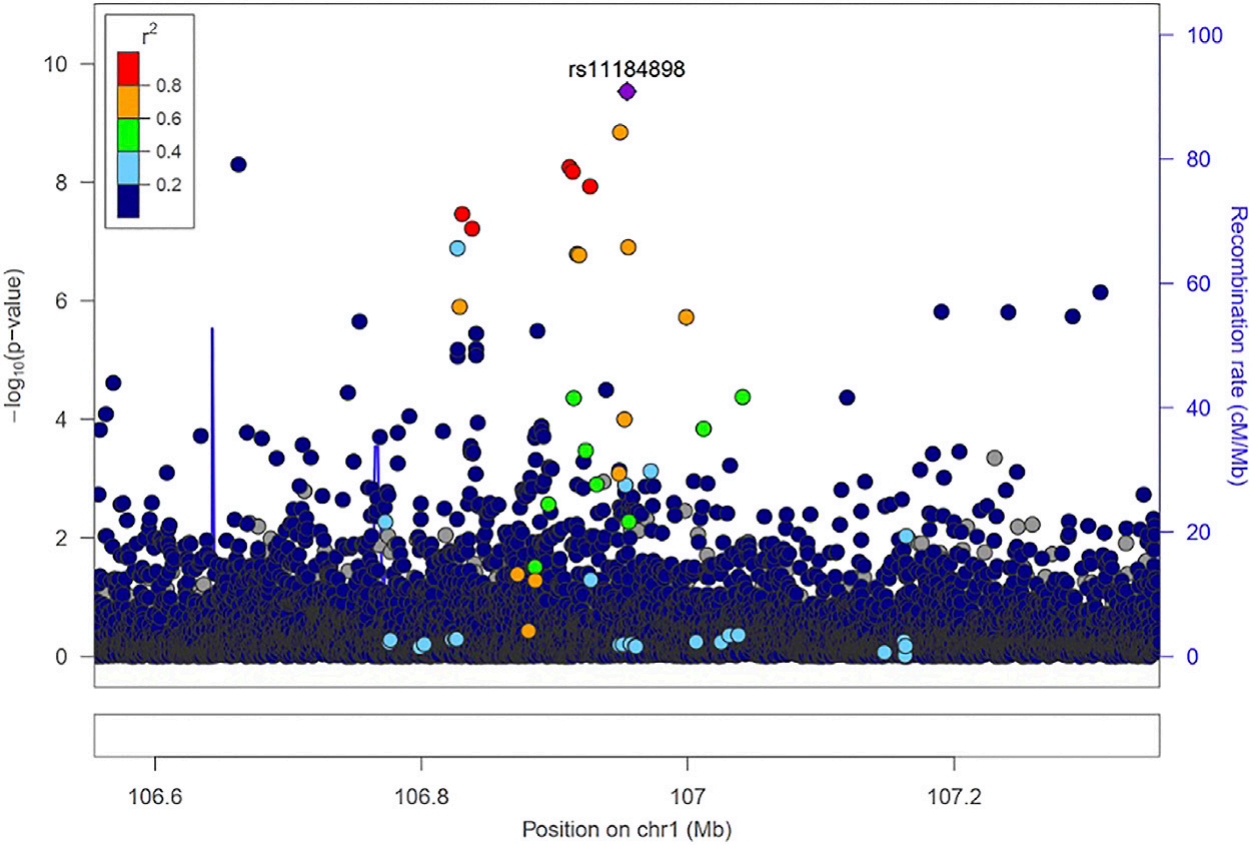
Locus zoom regional association plot for rs1184898.

Fine mapping seeks to analyze a trait-associated region to determine variants that are causal to a trait of interest. Fine mapping of loci was carried out within 250 kb upstream and downstream genomic distance of the sentinel variant identified *via* metanalysis. For each locus fine-mapped, we established the 99% credible set of SNPs that jointly make 99% of the posterior probability of driving the association (Figure 2). The fine-mapping analysis revealed that some of the lead variants such as rs369124352 (*MAGI3*), rs10918211 (*LRRC52*), and rs10800292 (*LINCO1363/POU2F1*) accounted for more than 50% of the posterior probability driving the association with these SNPs as the only variant within the 99% credible set. Several other variants were identified as the causal SNPs other than the sentinel SNP driving the association in WBC trait subtypes. Summary of the 99% credible set of variants driving the WBC trait can be found in Supplementary Table S2. We further went ahead and checked if these causal SNPs and their corresponding genes have been previously reported using the GWAS catalogue and Open Target; only five variants—rs11184898 (*LOC126987, MTCO3P14*) (Figure 3), rs907662 (*LINC01525*), rs7553527 (*GAPDHP32, HSD3BP3*), rs1923504 (*FLG-AS1, HMGN3P1*), and rs10733045 (*TRK-CTT13-1, MGST3*)—have not been reported to be associated with any WBC trait.

### Causal Effects of WBC Traits on Asthma

**FIGURE 4:**
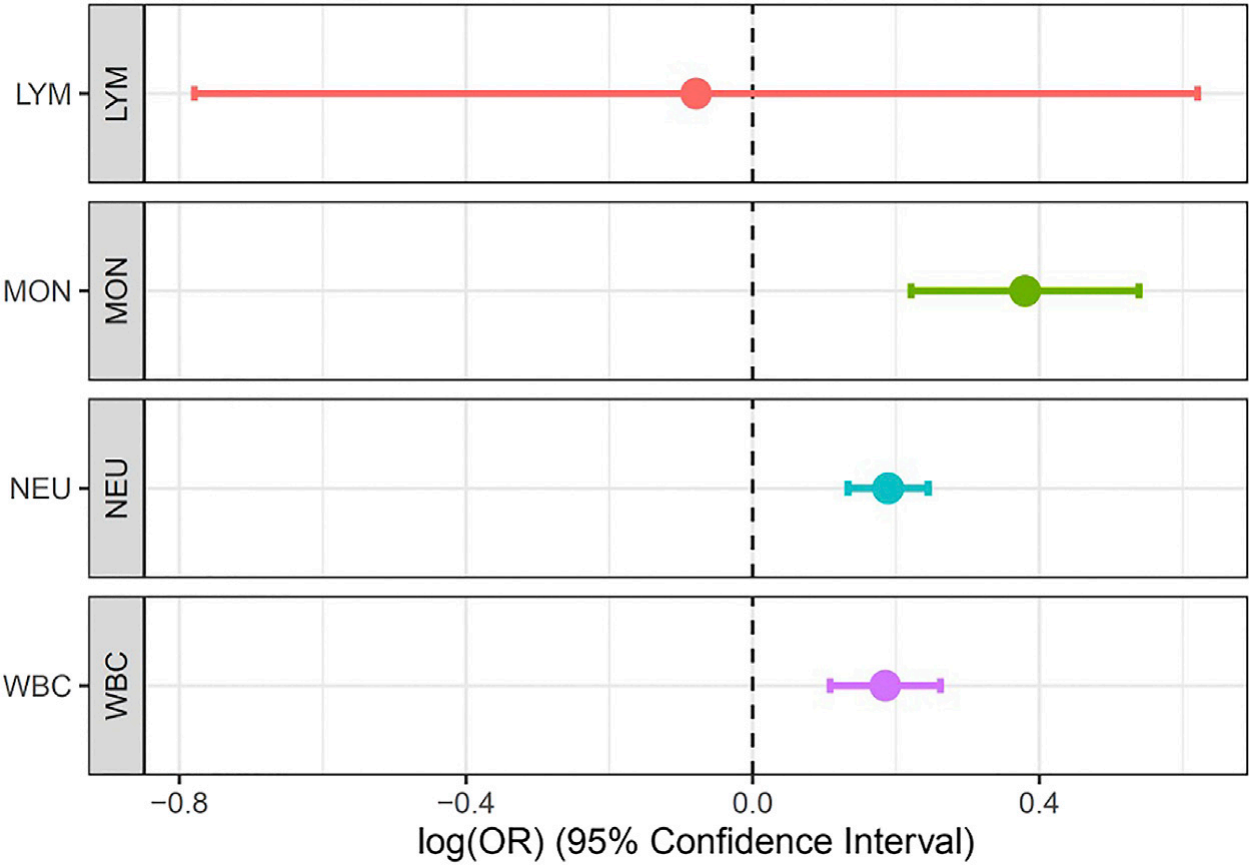
Forest plot for the association between Lym, Mon, Neu, and WBC with asthma estimated using MR-IVW method.

We set out to evaluate the causal relationship between WBC traits (Lym, Mon, Neu, and WBCc) and asthma. Our MR analysis identified strong positive association of Mon (IVW estimate = 0.38, CI: 0.221, 0.539, *p* < 0.001), Neu (IVW estimate = 0.189, CI: 0.133, 0.245, *p* < 0.001), and WBCc (IVW estimate = 0.185, CI: 0.108, 0.262, *p* < 0.001) with increased risk of asthma. However, there were no evidence of causal relationship between Lym and asthma risk (IVW estimate = −0.079, CI: −0.779, 0.621, *p* = 0.825) (Figure 4, Supplementary Table S3), though there was an inverse relationship between Lym and asthma risk. The causal estimates of the weighted median and the MR-egger methods of Mon showed a similar effect to the IVW method (Supplementary Table S3).

### Functional Analysis

**FIGURE 5:**
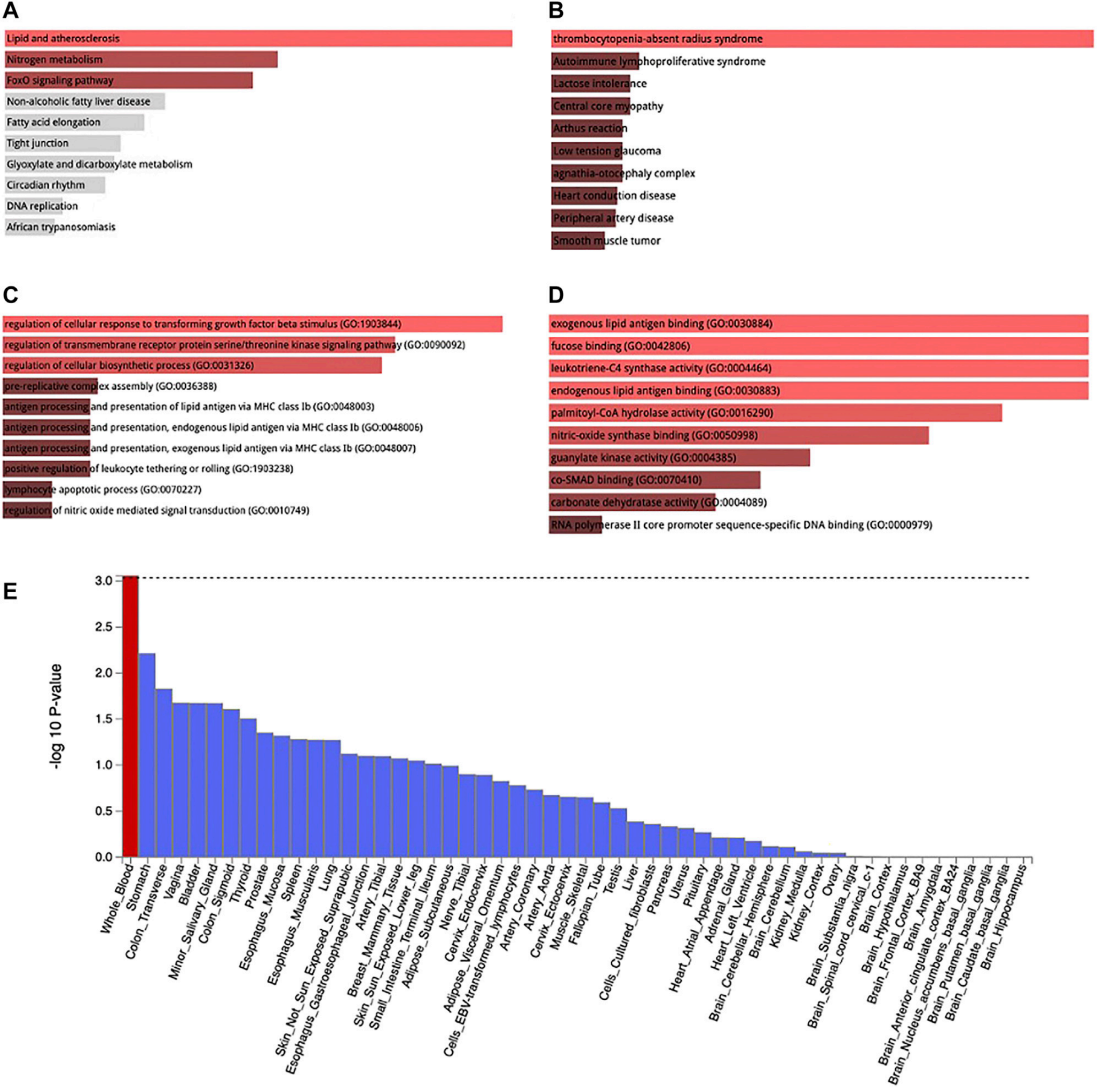
KEGG pathway enrichment **(A)**, Jensen disease enrichment **(B)**, Biological process **(C)**, and Molecular function **(D)** of the genes associated with WBC subtypes identified by metanalysis. Tissue expression of enriched genes **(E)**. Red bar in 3 E connotes tissues with significant enrichment while blue bars indicate tissues with no significant enrichment.

MAGMA analysis using GTEx v8: 54 tissue types and GTEx v8: 54 general tissue types showed significant expression in whole blood (Figure 5E). The loci were also expressed in other tissues such as stomach and the colon but not at a significant level. Lipid and atherosclerosis, nitrogen metabolism, and FoxO signaling pathway are some of the pathways enriched by these loci [Figure 5A, Supplementary Table S4 (ST1)]. These loci were mapped at a significant level to thrombocytopenia-absent radius syndrome, autoimmune lymphoproliferative syndrome, lactose intolerance, central core myopathy etc. [Figure 5B, Supplementary Table S4 (ST2)]. They are also involved in the regulation of cellular response to transforming growth factor beta stimuli, regulation of transmembrane receptor protein serine/threonine kinase signaling pathway, regulation of cellular biosynthetic process, etc. (Figure 5C), Supplementary Table S4 (ST3)] and also in exogenous lipid antigen binding molecular functions (Figure 5D).

## Discussion

To the best of our knowledge, this is the first study to explore the causal relationship of WBC traits and asthma in an African population. Using metanalysis and Bayesian fine mapping, we identified 269 significant SNPs associated with WBC traits. Among these, five genes (*LOC126987*/*MTCO3P14*, *LINC01525*, *GAPDHP32*/*HSD3BP3*, *FLG-*AS1, and *TRK-CTT13-*1/MGST3) for the first time are reported to be associated with WBC traits. We also found causal relationship between Mon, Neu, and WBCc with asthma, while no causal relationship was seen between Lym and Asthma.

*FLG-AS1* (FLG Antisense RNA 1) is an RNA gene that is a member of the long non-coding RNA (lncRNA). FLG-AS1 is associated with asthma (Ferreira et al., 2019; Johansson et al., 2019; Pividori et al., 2019; Zhu et al., 2019; Olafsdottir et al., 2020), melanoma (Rashkin et al., 2020), eczema (Johansson et al., 2019; Kichaev et al., 2019), and acute myeloid leukemia (Lv et al., 2017). Microsomal glutathione S-transferase 3 encoded by *MGST3* has been shown to help in cellular defense of host organisms against lipid hydroperoxides, which may arise as a result of oxidative stress (Jakobsson et al., 1997; Stamova et al., 2013). There are evidences of oxidative stress in asthma (Misso and Thompson, 2005; Andrianjafimasy et al., 2017; Sahiner et al., 2018), this may explain the enrichment of this gene in the blood and causal association with asthma.

Interestingly, our result is consistent with the multivariable MR analysis of Astle et al. (2016), which showed a protective effect of monocytes on Asthma. Furthermore, we also found a protective effect of neutrophils against asthma, consistent with Guyatt et al. (2020), which did not find substantial evidence for a harmful effect of neutrophils on asthma.

We found atherosclerosis to be significant in the KEGG pathway enrichment, and atherosclerosis is a major risk factor of coronary heart disease (CHD). Inflammation is an attribute of atherosclerosis; hence, several inflammatory cells such as Mon, Lym., Eos, and Neu have been implicated in CHD (Prentice et al., 1982; Madjid and Fatemi, 2013). Most importantly, several epidemiological studies have revealed that leukocyte count is an independent risk factor for CHD, and a risk factor for future cardiovascular events in individuals who do not have cardiovascular diseases (Weijenberg et al., 1996; Madjid et al., 2004; Chen et al., 2018). WBC count has also been suggested as a risk factor for atherosclerotic vascular diseases (Do LeeDo et al., 2001).

Significant enrichment of thrombocytopenia-absent radius syndrome (TAR), which is a rare congenital disorder (Greenhalgh et al., 2002), suggests the involvement of some WBC trait genes in the pathogenesis of this disease, as this disease is characterized by low levels of platelets in the blood (Greenhalgh et al., 2002).

Compared to other studies, one of the major strengths of this study is the use of African-ancestry data and the increased power from metanalysis. This enables the discovery of loci that have otherwise not been reported by previous studies. In addition, the CAAPA summary statistics is one of the latest cohorts of Asthma in Africa-admixed population.

We also applied different sensitivity analysis in our MR analysis, and we used strong instrumental variables by assessing the instrumental validity.

This study has its limitation, which is characteristic of two-sample MR analysis. Similar to any other non-experimental data that seek to make causal inference, where some experimentally unverifiable assumptions are made, our study is not an exception to this limitation.

Conclusively, we found evidence of causality between some WBC traits and asthma, and though some observational and MR data support these results, we believe more laboratorial experiments are needed to understand the biological mechanism of this causality.

## Key Messages

• We carried out a well-powered meta-analysis genome-wide association study of white blood cell (WBC) traits in African populations.
• We identified not previously known genes driving WBC traits in an African population.
• Bayesian fine mapping identified more credible variants with high posterior probability associated with WBC subtypes.
• Mendelian Randomization Analysis found a causal relationship between monocyte count, neutrophil count, and white blood cell count with asthma in African populations.
• Findings from this study could provide more insight into the roles WBC traits play in the pathogenesis of asthma and could as well provide some directions in the treatment of asthma.

## Data Availability

The original contributions presented in the study are included in the article/Supplementary Material. Further inquiries can be directed to the corresponding author.
